# Prevalence and Social Inequality in Experiences of Domestic Abuse Among Mothers of Young Children: A Study Using National Survey Data from Scotland

**DOI:** 10.1177/0886260520980392

**Published:** 2021-01-08

**Authors:** Valeria Skafida, Fiona Morrison, John Devaney

**Affiliations:** 1 University of Edinburgh, Scotland; 2 University of Stirling, Scotland

**Keywords:** mothers, children, domestic violence, coercive control, survey data, Scotland

## Abstract

Domestic abuse is a pernicious societal issue that has both short- and long-term consequences for those who are victimized. Research points to motherhood being linked to women’s victimization, with pregnancy being a particular point of risk. Across UK jurisdictions, new legislation aims to extend the criminalization of domestic abuse to include coercive control. Less clear is the relationship between mothers’ victimization of different “types” of abuse and other factors such as age, socioeconomic status, and level of education. The article makes an original contribution to knowledge by addressing these limitations of the existing literature. Using nationally representative data from a Scottish longitudinal survey (*N* = 3,633) into children’s development this article investigates the social stratification of mothers’ exposure to different types of abuse, including coercive control, physical abuse, and threats. Overall, 14% of mothers report experiencing any type of domestic abuse since the birth of the study child (age 6), of which 7% experienced physical abuse. Compared to mothers in the highest income households, mothers in the lowest income quintile were far more likely to experience any form of abuse (Logistic Regression, *OR* = 3.55), more likely to have experienced more types of abuse and to have experienced these more often (*OR* = 5.54). Age had a protective effect, with mothers aged 20 or younger at most risk of abuse (*OR* = 2.60 compared to mothers aged 40+). Interaction effects between age and income suggested that an intersectional lens may help explain the cumulative layers of difficulty which young mothers on low incomes may find themselves in when it comes to abusive partners. The pattern of social stratification remained the same when comparing different types of abuse. Mothers of boys were more likely to experience abuse, and to experience more types of abuse, more often. We reflect on how these findings could inform existing policy interventions.

## Introduction

Domestic abuse is a pernicious societal issue that has both short- and long-term consequences for those who are victimized. According to the UK Office for National Statistics data, 26.2% of adult women experience some form of domestic abuse in their lifetime (Office for National Statistics, 2018). Research points to motherhood as being linked to women’s victimization, with pregnancy being a particular point of risk both for the onset and the escalation of domestic violence (Office for National Statistics, 2018; O’Reilly, 2007). There are no official statistics on children living with domestic abuse, though this is estimated to be 1 in 5 of all children (Radford, 2011). However, there is robust evidence to highlight that children living in households with domestic abuse are significantly affected by this exposure (Holt et al., 2008; McTavish et al., 2016).

Across jurisdictions, we see the introduction of new legislation that aims to extend the criminalization of domestic abuse to include coercive control, reflecting survivors accounts of domestic abuse. Legislation introduced in 2015 for England and Wales criminalized coercive or controlling behavior towards partners/spouses, and a draft Domestic Violence and Abuse Bill was published in March 2020. In Scotland, the Domestic Abuse (Scotland) Act 2018 incorporates physical, emotional, and psychological violence into one continuing offence. According to the latter, the legal definition of domestic abuse now also includes the following:

behaviour directed at [victim],^1^ 1The legislation refers to the victim as “B” and perpetrator as “A”, and these terms have been replaced with victim and perpetrator respectively, to aid in interpretation. The legislation can be found at https://www.legislation.gov.uk/asp/2018/5/section/2/enacted at child of [victim] or at another person, that either (i) has as its purpose one or more of the relevant effects—or (ii) would be considered by a reasonable person to be likely to have one or more of the relevant effects: (a) making the [victim] dependent on, or subordinate to the [perpetrator]; (b) isolating [the victim] from friends, relatives or other sources of support; (c) controlling, regulating or monitoring [the victim’s] day-to-day activities; (d) depriving [victim] of, or restricting [victim’s], freedom of action; (e) frightening, humiliating, degrading or punishing [victim].

According to a literature review by O’Reilly (2007), there is a paucity of literature on domestic abuse against women in their childbearing years, and particularly in the postnatal period. Most prevalence studies using national samples focus on women using a very wide age-range, and often enquiring about abuse across a lifetime (Alhabib et al., 2010; Capaldi et al., 2012; O’Reilly, 2007). This makes it harder to determine how many parents and how many children are victims of abuse. In terms of the social stratification of abuse experiences, international research to date suggests that while no socioeconomic group is immune, domestic abuse experiences are stratified by socioeconomic factors (Afzal et al., 2018; Alhabib et al., 2010; Capaldi et al., 2012). However, according to a review of prevalence studies, while many studies control for demographic variables in their analyses, few of them actually focus on these as predictors (Capaldi et al. 2012), and comparing results across studies is also challenging due to documentation of such predictors being poorly reported (Alhabib et al., 2010). Looking across a range of studies, the review by Capaldi et al. (2012) concludes that income and unemployment variables are stronger predictors of domestic abuse than education variables, but these measures are rarely looked at in conjunction within single-study designs. Existing evidence concludes that age is a protective factor against domestic abuse. However, a weakness of the existing prevalence literature is how interaction effects between different socioeconomic predictors of domestic abuse experiences, such as age and income, are either rarely explored or mentioned. Some papers do explore interaction effects between other predictive factors, such as family and school disadvantage (Spriggs et al., 2009), or between age and gender for studies of both male and female perpetrators (Capaldi et al., 2007).

Additionally, most existing prevalence studies do not look at a range of abusive behaviors including coercive control, which reflect contemporary understandings of domestic abuse that have gained increasing traction in research, discourse, and now criminal legislation. Scottish Women’s Aid, the leading third sector organization working towards the prevention of domestic abuse in Scotland gives the following examples of coercive behavior^2^ 2
https://womensaid.scot/information-support/what-is-domestic-abuse/
: isolating someone from friends and family; depriving someone of basic needs, such as food; monitoring someone’s time; monitoring someone via online communication tools or spyware; taking control over aspects of someone’s everyday life, such as where they can go, who they can see, what they can wear and when they can sleep; depriving someone access to support services, such as medical services; repeatedly putting someone down, such as saying they are worthless; humiliating, degrading or dehumanizing someone; controlling someone’s finances; making threats or intimidating someone. Research on domestic abuse has discussed the importance of recognizing such forms of abuse, and in the literature this is often referred to as psychological abuse (see Kelly, 2004 for a review). More recent work has also sought to theorize such abuse, and the term coercive control as a label to encompass such behaviors was coined by Stark (2007).

In a review of global prevalence studies on domestic abuse, out of 155 studies included, 88 were population-based studies; of these, 24 asked about physical, sexual, and emotional abuse, and of these, 8 used a random sample greater than 1,000 participants (Alhabib et al., 2010). Overall, studies on large nationally representative surveys that examine different forms of abuse are few and far between. Furthermore, there is also a dearth of studies which *compare* the prevalence of different forms of violence to each other. For example, only 2 of 228 papers in a review of prevalence studies by Capaldi et al. (2012) look at differences in exposure to different types of abuse, with one focusing on aggression of female soldiers towards male spouses (Newby et al., 2003) and the other on adolescent perpetrators and teenage dating violence (Foshee et al., 2009).

Our study makes an original contribution to knowledge through addressing some of the common aforementioned limitations in the existing literature. We use a nationally representative social survey to look at the prevalence of domestic abuse in a broadly defined sense, looking not only at physical violence but also psychological forms of abuse. We focus on mothers of young children and on abuse experienced since motherhood, and we aim to get a comprehensive understanding of whether and in what ways the prevalence of different forms of abuse varies for different groups of mothers. We also explore the presence of interaction effects between different socioeconomic characteristics and explore the implications of this for discussions regarding the socially stratified nature of domestic abuse. Ultimately, we hypothesize that (a) there will be a social gradient to experiences of domestic abuse; (b) mothers from more disadvantaged backgrounds will be more likely to experience any and all types of abuse, and to experience this more often; (c) multiple dimensions of disadvantage will correlate with a cumulatively higher risk of experiencing domestic abuse.

## Methods

### Dataset Description

Growing Up in Scotland (GUS) is a longitudinal nationally representative prospective study of preschool children and their families in Scotland. It is the only longitudinal dataset of a UK nation to have questions on domestic abuse asked of mothers of young children. The cohort used in this analysis consisted, at the first survey, of 5,217 babies born between June 2004 and May 2005. Babies were c.10 months old at the time of the first sweep. Interviews were carried out in participants’ homes usually with the child’s mother, and mothers and partners (if present) were asked a range of questions about themselves and their children’s development in one-to-one interviews using Computer Assisted Personal Interviewing (CAPI) software. The stratified random sample was drawn from a record of Child Benefit claimants, a universal social welfare payment virtually all families were eligible to, and which has an estimated 97% coverage of the eligible population. Attrition rates are low for a survey of this nature (at 87% response rate of surveys issued, and 70% response rate based on sample at sweep 1). The official user guide for the first sweep of data describes the survey design in further detail (Corbett et al., 2007). GUS received ethics approval by the Scotland “A” MREC committee, and the research reported in this article received ethical approval from the University of Edinburgh’s School of Social and Political Science ethics committee. For the purpose of this study, 3,646 mothers were part of the 6th survey sweep, when children were 6 years old, and 3,633 had valid data on all relevant variables in the analysis (7 had missing data on age; 5 on education; 1 on ethnic background).

### Dependent Variables

The main outcome of interest in this article, maternal experience of domestic abuse, is recorded when children approached their 6th birthday, and the survey ran annually from when children were aged 10 months old. The domestic abuse module, unlike the majority of the survey which was interviewer led, was a self-complete feature which mothers could fill in privately. Mothers were asked to report if they had experienced a range of different types of violence, which broadly speaking reflect coercive control, threats, and physical and sexual violence. The questionnaire for this module was based on the questions used in the Scottish Crime and Justice Survey at the time of the GUS questionnaire design. The key difference is that GUS asks about experiences of such abuse in a 6-year period from the birth of the study child to the present day, whereas the Scottish Crime and Justice Survey asks respondents to report on violence occurring in the last 12 months. Table 1 shows the wording of each original question and also the ways in which these were recoded for analysis. Using these questions, we developed three categories of dependent variables as described below, looking at any incidence of abuse, at different frequencies of any abuse, and at different types of abuse.

**Table 1.** Wording of Original Survey Questions on Domestic Abuse and Recoded Variables.

Survey Questions and Deposited Derived Variables	Study Recoded Variables
In the time since child was born, has any partner or ex-partner ever done any of the following things to you? [yes/no]	
Stopped you having a fair share of the household money or taken money from you	Experience of coercive control [if answered yes to any of these questions]
Repeatedly put you down so that you felt worthless	
Behaved in a jealous or controlling way, for example, restricting what you can do, who you can see, what you can wear	
Pushed you or held you down	Experience of physical violence [if answered yes to any of these questions]
Kicked, bitten or hit you	
Choked or tried to strangle/smother you	
Used a weapon against you, for example, an ashtray or a bottle	
Forced you or tried to force you to take part in any sexual activity when you did not want to	
Threatened to hurt you	Experience of threats [if answered yes to any of these questions]
Threatened to hurt someone close to you, such as your children, family members, friends, or pets	
Threatened to, attempted to, or actually hurt themselves as a way of making you do something or stopping you from doing something	
Threatened to kill you	
	Experience of any violence [if mother answered yes to any of the original questions].
How many times since child was born have any of these things happened to you?	Abuse intensity scale and categorical variable [described in text]
Number of types of abuse experienced (deposited in survey)	

#### Any abuse.

This dependent variable is coded as 1 for all mothers who answered “yes” to any question about abuse (*N* = 435).

#### Abuse intensity.

Using information on how many different types of abuse a mother had experienced, and on how often the abuse had occurred, a standardized scale was created (alpha: 0.65). Based on this scale, a categorical variable for domestic abuse intensity was then created to differentiate between category 0 (no abuse); 1 (abuse, low intensity: the bottom 50% of the distribution); 2 (abuse, high intensity: the top 50% of the frequency distribution). In brief, mothers in category 1 would generally report having experienced fewer types of abuse and on fewer occasions, and vice versa for mothers in category 2. We are aware of potential limitations of any abuse intensity measure which is relying on counting incidents of abuse events. We discuss these in detail in the limitations section.

#### Different types of abuse.

Using the conceptual structure of the survey questionnaire, we also look at (Table 6) differences in experiences of each of the types of violence enquired about, which in the questionnaire were differentiated as 1: coercive control; 2: threats of abuse; 3: physical abuse (including sexual). Though an argument could have been made for looking at sexual abuse separately, sample size for this particular response (*N* = 35) was too low for meaningful multivariate analysis.

### Independent Variables

Maternal social class status was controlled for, and mothers were categorized into social class categories according to the National Statistics Socio-Economic Classification (NS-SEC) scheme.^3^ 3Office for National Statistics; the National Statistics Socio-economic classification (NS-SEC). https://www.ons.gov.uk/methodology/classificationsandstandards/otherclassifications/thenationalstatisticssocioeconomicclassificationnssecrebasedonsoc2010 This is calculated on the basis of information regarding the individual’s working conditions, job security, timing of payments, opportunities for promotion, and incremental pay (Rose & O’Reilly, 1998). A banded variable indicating the mother’s highest educational qualifications was used for the analysis to represent the mother’s educational level. Income data were obtained by asking the mother to select one of 17 income bands that reflected total household income before tax. Equivalized income was calculated using the Organisation for Economic Co-operation and Development (OECD) modified equivalence scales and procedure (Chanfreau & Burchardt, 2008). The mother’s age at the time of birth of the sample child was also controlled for, as well as the sex of the study child. Due to relatively low proportions of ethnic minorities in Scotland the GUS survey only provides a derived binary ethnicity question (white vs. other background) which was also controlled for.

### Statistical Analysis

Binary logistic regression (Table 4) was specified to explore exposure to any abuse, where the model predicts if the mother has experienced domestic abuse by any partner since the birth of the study child (i.e., in the last 6 years). Multinomial logistic regression models were specified to compare relative risk ratios of experiencing low and high intensity abuse compared to the no abuse reference category (Table 4). Interaction effects were tested between socioeconomic indicators and ethnicity, but these were not significant. In preliminary models, not shown, interaction effects were also tested between the ethnic background and socioeconomic variables, but these too were nonsignificant. Significant interaction effects between household income and age, and between income and maternal education are reported in Table 5. For this table, income, age, and education variables had to be recoded into binary variables for Stata to be able to calculate predicted probabilities, due to low sample sizes when using the original coding of these variables. The coefficients of the other variables in the models remained largely unchanged after controlling for interaction effects (results not shown). Table 6 compared the predicted probabilities for experience of different types of abuse. Comparisons based on predicted probabilities are preferred as comparisons on the basis of odds ratios are invalid albeit commonly seen in published research (Mood, 2010). Here, each model displays the predicted probability of exposure to (a) coercive control, (b) threats of abuse, and (c) physical violence. Multicollinearity tests showed that none of the independent variables in the regression analyses reached the commonly used threshold of <0.200 (Menard, 1995). Appropriate sample weights were used for the analysis to adjust for nonrandom nonresponse bias, and for unequal probability of selection for some children. Stata version 15.0 was used for all analyses.

## Results

Table 2 shows the incidence rate for exposure to all types of domestic abuse and different types of domestic abuse for mothers. Circa 14% report exposure to abuse since the birth of the study child. Since the birth of the study child most mothers who did experience some form of abuse experienced one type (43%) while just under 60% experienced two or more types (percentages derived, not shown). Among mothers experiencing abuse, c.60% reported this occurring between 1 and 6 or more times in the 6 years since the birth of the child (c.19% experienced such abuse just once, figures not shown). Approximately 15% of mothers reported this happening too many times to count, though this excludes 26% of mothers who did not remember, did not know or did not wish to answer the frequency question. Looking at the differences in exposure to different types of abuse, the most commonly experienced form of abuse is coercive control. This form of abuse has only recently been recognized in criminal legislation in the different UK countries, which is a significant improvement in the legislative structure given how prevalent it is.

**Table 2.** Descriptive Statistics: Percentages (%) and Confidence Intervals (CI) for Exposure to Different Types of Domestic Abuse Since Birth of Sample Child (*N* = 3,646).

Weighted Data ^a^	%	95% CI
*Experience of number of types of violence*		
None	86.4	[85.1–87.6]
One	5.8	[5.1–6.6]
Two	2.5	[1.9–3.1]
Three or more	5.4	[4.6–6.3]
*How many times since child was born have any of these things happened to you?*		
One to three times	43.3	[43.3–43.3]
Four to five, or 6 or more times	16	[16.0–16.0]
Too many to count	15.2	[15.2–15.2]
Don’t know/remember, answer refused	25.5	[25.5–25.5]
*Since the birth of the sample child (current or previous partner)*		
Experience of any coercive control	10.6	[9.6–11.8]
Experience of any physical violence	7.2	[6.2–8.2]
Experience of any threats	6.6	[5.6–7.7]
Experience of any violence (any of the above)	13.6	[12.4–14.9]
Experience of any violence from current partner	3.5	[3.0–4.2]
*Scottish Crime and Justice Survey 2014–2015^a^*		
Women experiencing partner abuse in last 12 months	3.4	N/A
*Note.* ^a^ Scottish Crime and Justice Survey data presented for comparison (Source: https://beta.gov.scot/publications/domestic-abuse-recorded-police-scotland-2016-17/pages/6/).		

Table 3 lays out a bivariate analysis of how different maternal and family characteristics relate to maternal exposure to all types of abuse. All independent variables, except ethnic background, show statistically significant differences in exposure. With regards to income and social class (NS-SEC), it appears mothers who are from disadvantaged backgrounds report a higher incidence of abuse than their more advantaged counterparts, and the incidence of abuse rises incrementally as income poverty increases. For example, 24% of mothers in the lowest income quintile compared to 6% of those in the highest income quintile report experiencing any abuse. Differences in abuse by maternal education do not follow such a clear-cut pattern, nor an incremental one, though those with no qualifications are the group most likely to report exposure to any abuse (17.5%).

**Table 3.** Bivariate Analysis: Proportions (%) and Confidence Intervals (CI) of Mothers Experiencing Any Domestic Violence by Mother and Child Characteristics (*N* = 3,633).

Weighted Data ^a^	%	95% CI	Chi^2^
*Maternal education*			*p* < .001
Degree or equivalent	10.1	[8.3–12.2]	
Vocational qualifications	15.5	[13.7–17.4]	
Higher grade or equivalent	7.8	[4.9–12.3]	
Standard grade	15.1	[11.4–19.8]	
No qualifications	17.5	[12.6–23.6]	
*Equivalized income*			*p < .001*
1st quintile	24.2	[21.0–27.6]	
2nd quintile	14.5	[11.9–17.5]	
3rd quintile	12.5	[9.9–15.7]	
4th quintile	6.4	[4.8–8.3]	
5th quintile	6.3	[4.6–8.4]	
Missing income data	9.3	[6.0–14.2]	
*Maternal NS-SEC*			*p < .001*
Managerial and professional	8.6	[7.4–10.0]	
Intermediate	14.5	[11.3–18.3]	
Small employers and own account holders	10.6	[7.1–15.4]	
Lower supervisory and technical	15.6	[10.6–22.4]	
Semi-routine and routine	24.1	[21.3–27.2]	
Never worked	21.9	[10.8–39.4]	
*Mother’s age at birth of sample child*			*p < .001*
Under 20	28.7	[21.4–37.4]	
20–29	16.1	[14.3–18.0]	
30–39	9.3	[8.0–10.7]	
40 or older	9.1	[5.2–15.3]	
*Mother’s ethnic background*			*p* = 0.140
White	13.7	[12.5–15.0]	
Other ethnic background	9.1	[5.1–15.7]	
*Number of children in the household*			*p* < .001
1	18.2	[15.1–21.8]	
2	10.9	[9.4–12.5]	
3	13.7	[10.9–17.1]	
4 or more	19.2	[14.3–25.3]	
*Child’s gender*			
Male	14.7	[13.0–16.7]	*p* = 0.024
Female	12.2	[10.9–13.7]	

The group most likely to experience abuse seems to be mothers aged 20 or younger when the study child was born (29%) in contrast to those who were 40 years or older (9%). It appears that mothers from a “white” ethnic background are more likely than other ethnic groups to report experiences of abuse, though the difference is not significant. The number of dependent children a mother has was correlated with experiencing abuse, but the pattern appears to follow a U-shaped curve. Mothers with only one child, and those with four children were more likely to experience abuse compared to those with two or three children. Finally, it seems mothers of boys were slightly more likely to report abuse (14.7%) compared to mothers of girls (12.2%), though it is important to remember that sex refers to the study child, and some mothers will have other children of the other genders as well in the home.

### Any Abuse

Table 4 lays out the results of the logistic and multinomial regression analyses. For exposure to any abuse, mothers from the lowest household income quintiles have a more than threefold chance of experiencing abuse compared to mothers from the highest income quintile (*OR* = 3.55). The trends for social class and education are not quite as straightforward to interpret. It seems mothers who are in routine and semi-routine occupations are more likely to experience any abuse (*OR* = 1.64) compared to those in managerial and professional occupations. As the descriptive statistics indicated, young mothers were more likely to be victims of abuse, and mothers who were aged 20 or younger at the birth of the study child had a higher chance of experiencing different types of violence (*OR* = 2.60). Mother’s ethnicity was not significant, and mothers with only one child (despite controlling for maternal age) were more likely to experience abuse compared to those with more children. Finally, mothers whose study children were boys were more likely to experience any abuse compared to mothers of girls (*OR* = 1.22). Maternal education, when compared to the descriptive statistics, appeared to tell a different story. After controlling for confounders strongly correlated with maternal education, mothers with below-degree level education were all *less* likely to report experiencing any abuse (*OR* ranges 0.39–0.56). We explore why this may be the case below.

**Table 4.** Logistic and Multinomial Regression: Experience of Any Abuse and Different Abuse Exposures.

	Logistic Regression (Ref. Group: No Abuse)		Multinomial Logistic Regression(Ref. Group: No Abuse)			
	Any Abuse		Any Abuse: Lower Intensity		Any Abuse: Higher Intensity	
	Odds Ratio^1^	95% CI^1^	Relative Risk Ratio	95% CI	Relative Risk Ratio	95% CI
*Maternal education*		
Degree or equivalent	1.00	[1.00, 1.00]	1.00	[1.00, 1.00]	1.00	[1.00, 1.00]
Vocational qualifications	0.81	[0.58, 1.13]	0.84	[0.55, 1.28]	0.78	[0.49, 1.23]
Higher grade or equivalent	0.39^**^	[0.21, 0.74]	0.43†	[0.18, 1.02]	0.36^*^	[0.15, 0.84]
Standard grade	0.55^*^	[0.35, 0.87]	0.39^**^	[0.21, 0.71]	0.67	[0.40, 1.14]
No qualifications	0.56^*^	[0.33, 0.94]	0.80	[0.41, 1.54]	0.38^**^	[0.19, 0.74]
*Equivalized income*						
1st quintile	3.55^***^	[2.23, 5.67]	2.12^*^	[1.11, 4.04]	5.54^***^	[2.86, 10.74]
2nd quintile	2.05^**^	[1.29, 3.26]	1.61	[0.85, 3.02]	2.61^**^	[1.37, 4.96]
3rd quintile	1.90^**^	[1.22, 2.96]	1.93^*^	[1.08, 3.44]	1.84	[0.88, 3.87]
4th quintile	1.01	[0.65, 1.57]	1.16	[0.66, 2.06]	0.85	[0.41, 1.73]
5th quintile	1.00	[1.00, 1.00]	1.00	[1.00, 1.00]	1.00	[1.00, 1.00]
Missing income data	1.33	[0.72, 2.47]	1.25	[0.57, 2.74]	1.36	[0.53, 3.50]
*Maternal NS-SEC*						
Managerial and professional	1.00	[1.00, 1.00]	1.00	[1.00, 1.00]	1.00	[1.00, 1.00]
Intermediate	1.20	[0.86,1.68]	1.01	[0.62, 1.65]	1.34	[0.81, 2.22]
Small employers and own account holders	0.88	[0.54, 1.42]	1.10	[0.60, 2.04]	0.71	[0.37, 1.35]
Lower supervisory and technical	1.31	[0.85, 2.03]	1.58	[0.89, 2.79]	1.11	[0.63, 1.96]
Semi-routine and routine	1.64^**^	[1.15, 2.36]	1.97^**^	[1.24, 3.13]	1.42	[0.86, 2.33]
Never worked	1.36	[0.55, 3.35]	1.03	[0.24, 4.48]	1.51	[0.52, 4.41]
*Mother’s age at birth of sample child*						
Under 20	2.60^**^	[1.30, 5.21]	2.42†	[0.95, 6.17]	2.68^*^	[1.03, 7.02]
20–29	1.66	[0.85, 3.23]	1.69	[0.68, 4.18]	1.62	[0.68, 3.86]
30–39	1.15	[0.59, 2.28]	1.12	[0.47, 2.67]	1.18	[0.46, 3.02]
40 or older	1.00	[1.00, 1.00]	1.00	[1.00, 1.00]	1.00	[1.00, 1.00]
*Mother’s ethnic background*						
White	1.00	[1.00, 1.00]	1.00	[1.00, 1.00]	1.00	[1.00, 1.00]
Other ethnic background	0.64	[0.32, 1.29]	0.75	[0.30, 1.87]	0.54	[0.23, 1.28]
*Number of children in the household*						
1	1.00	[1.00, 1.00]	1.00	[1.00, 1.00]	1.00	[1.00, 1.00]
2	0.65^**^	[0.48, 0.88]	0.57^**^	[0.40, 0.80]	0.74	[0.48, 1.15]
3	0.73†	[0.51, 1.04]	0.64^*^	[0.41, 1.00]	0.82	[0.51, 1.33]
4 or more	0.91	[0.60, 1.39]	1.03	[0.60, 1.77]	0.79	[0.42, 1.51]
*Child’s gender*						
Male	1.22^*^	[1.01, 1.48]	0.93	[0.70, 1.22]	1.57^***^	[1.22, 2.03]
Female	1.00	[1.00, 1.00]	1.00	[1.00, 1.00]	1.00	[1.00, 1.00]
*N*	3,634		3,634			

*Note.* ^1^Exponentiated coefficients; 95% confidence intervals in brackets.

^†^*p* < .1, ^*^p < .05, ^**^p < .01, ^***^p < .001.

Table 5 shows the significant interaction effects found between household income and maternal age for the experience of any domestic abuse. These results suggest that while both low income and a young age at the birth of the sample child correlate with higher chances of experiencing violence, mothers who were *both* under 20 and on the lowest income quintile had a significantly much higher chance of experiencing domestic abuse, with a predicted prevalence of 34% (1 in 3 mothers) and relative odds of 4 to 1 compared to mothers aged 20 or older who were not in the bottom income quintile. Table 5 also shows the interaction effects between maternal education and household income and goes some way in explaining the aforementioned reversal of the relationship between maternal education and reporting of domestic abuse after controlling for socioeconomic confounders. It seems that mothers in the poorest income households who have the highest educational qualifications are also significantly more likely to experience abuse than other groups (predicted prevalence of 29.5% compared to a prevalence of 10.8%–18.3% in the other 3 groups). We queried whether this effect could be explained by controlling for the characteristics of the partners that women were with and ran a logistic regression (not shown) on a subsample of mothers (*N* = 2,575) who had been with the same partner across all sweeps, where we controlled for the partner’s educational qualifications and his social class (NS-SEC). Even with these controls the relationship between maternal education and abuse remained unchanged, and mothers with fewer qualifications were still significantly less likely to report experiences of abuse.

**Table 5.** Interaction Effects for Age and Income: Predicted Prevalence and Confidence Intervals (CI) of Any Experience of Domestic Abuse (*N* = 3,633).

	Predicted Prevalence^b^ and 95% CI		Relative Odds	
Weighted data	Bottom Income Quintile	Income Quintiles 2, 3, 4, 5^a^	Bottom Income Quintile	Income Quintiles 2, 3, 4, 5^a^
Aged under 20	34.0% (22.8–45.1)	12.6% (4.9–20.2)	4.30	1.20
Aged 20 or older	18.1% (14.8–21.3)	10.7% (9.4–12.0)	1.84	1 (Ref)
				
Maternal education: Degree or above	29.5% (16.5–42.5)	12.6% (10.2–15.1)	3.44	1.19
Maternal education: Below degree	18.3% (15.2–21.3)	10.8% (9.2–12.4)	1.84	1 (Ref)

*Note.* ^a^Includes missing income data.

^b^Controlling for full list of confounders as per Table 4 logistic regression models.

### Abuse Intensity

Based on the multinomial logistic regression results (Table 4), the sociodemographic trends and patterns for the different abuse intensities, as specified in this study, seemed to follow the patterns already described above. A notable insight from comparing prevalence of different intensities of abuse is that the gradient of unequal exposure to high-intensity abuse was larger than for low-intensity abuse when looking at household income. So, for example, compared to the highest income households, mothers on the lowest incomes had a twofold chance of experiencing low-intensity abuse and a fivefold chance of experiencing high-intensity abuse. The second noteworthy insight is that the aforementioned difference in experiencing abuse depending on the study child’s sex seems to be driven by experiences of mothers in the high-intensity abuse category. Thus, compared to having a female study child, and not accounting for the gender of any other children in the home, having a male study child is linked to 1.53 higher odds of experiencing high-intensity abuse (whereas the coefficient is nonsignificant for the low-intensity abuse group).

### Different Types of Abuse

Table 6 shows differences in predicted probability of experiencing different types of violence. As above, the patterns of prevalence follow the patterns that have been discussed thus far. That is, for each different type of abuse, mothers on lower household incomes, lower social class categories, younger ages and with higher educational qualifications had a relatively higher predicted probability of experiencing each type of abuse. Notable differences are that more coefficients reached statistical significance for coercive control (12 coefficients) than for other types of abuse (8 and 5 coefficients), and this is most likely driven by the higher sample size for this category of abuse compared to the others.

**Table 6.** Logistic Regression Predicted Prevalence (PP) and Confidence Intervals (CI) for Experience of Different Types of Abuse.

	Coercive Control(*N* = 331/3,533)		Threats(*N* = 182/3,384)		Physical Abuse(*N* = 220/3,422)		
	PP	95% CI	PP	95% CI	PP	95% CI	
*Maternal education*							
Degree or equivalent	0.00	[0.00, 0.00]	0.00	[0.00, 0.00]	0.00	[0.00, 0.00]	
Vocational qualifications	–0.01	[–0.04, 0.02]	–0.01	[–0.04, 0.02]	–0.01	[–0.04, 0.01]	
Higher grade or equivalent	–0.07^***^	[–0.10, –0.03]	–0.04^**^	[–0.07, –0.02]	–0.04^**^	[–0.07, –0.01]	
Standard grade	–0.04^*^	[–0.08, –0.01]	–0.01	[–0.04, 0.02]	–0.02	[–0.05, 0.01]	
No qualifications	–0.04^*^	[–0.08, –0.00]	–0.02	[–0.05, 0.01]	–0.03	[–0.06, 0.01]	
*Equivalized income*							
1st quintile	0.12^***^	[0.07, 0.16]	0.08^***^	[0.04, 0.13]	0.08^***^	[0.04, 0.12]	
2nd quintile	0.05^**^	[0.01, 0.08]	0.03^*^	[0.00, 0.05]	0.03^*^	[0.00, 0.06]	
3rd quintile	0.04^*^	[0.01, 0.07]	0.02	[–0.01, 0.04]	0.01	[–0.01, 0.04]	
4th quintile	–0.01	[–0.03, 0.02]	0.00	[–0.02, 0.02]	–0.01	[–0.03, 0.02]	
5th quintile	0.00	[0.00, 0.00]	0.00	[0.00, 0.00]	0.00	[0.00, 0.00]	
Missing income data	0.02	[–0.02, 0.06]	0.00	[–0.03, 0.03]	–0.01	[–0.04, 0.02]	
*Maternal NS-SEC*							
Managerial and professional	0.00	[0.00, 0.00]	0.00	[0.00, 0.00]	0.00	[0.00, 0.00]	
Intermediate	0.01	[–0.02, 0.04]	0.03^*^	[0.00, 0.05]	0.02	[–0.01, 0.04]	
Small employers and own account holders	–0.01	[–0.04, 0.03]	–0.00	[–0.02, 0.02]	–0.02	[–0.05, 0.01]	
Lower supervisory and technical	0.03	[–0.01, 0.07]	0.00	[–0.02, 0.03]	0.02	[–0.02, 0.05]	
Semi-routine and routine	0.04^*^	[0.00, 0.09]	0.02†	[–0.00, 0.04]	0.03†	[–0.00, 0.06]	
Never worked	0.05	[–0.05, 0.14]	0.04	[–0.03, 0.10]	0.01	[–0.06, 0.08]	
*Mother’s age at birth of sample child*							
Under 20	0.09^**^	[0.03, 0.15]	0.05^*^	[0.00, 0.10]	0.09^*^	[0.02, 0.15]	
20–29	0.04^*^	[0.00, 0.09]	0.01	[–0.02, 0.04]	0.03	[–0.01, 0.07]	
30–39	0.02	[–0.03, 0.06]	–0.01	[–0.04, 0.03]	0.01	[–0.03, 0.05]	
40 or older	0.00	[0.00, 0.00]	0.00	[0.00, 0.00]	0.00	[0.00, 0.00]	
*Mother’s ethnic background*							
White	0.00	[0.00, 0.00]	0.00	[0.00, 0.00]	0.00	[0.00, 0.00]	
Other ethnic background	–0.04^*^	[–0.07, –0.00]	–0.04^***^	[–0.06, –0.02]	–0.01	[–0.06, 0.03]	
*Number of children in the household*							
1	0.00	[0.00, 0.00]	0.00	[0.00, 0.00]	0.00	[0.00, 0.00]	
2	–0.04^**^	[–0.07, –0.01]	–0.01	[–0.04, 0.01]	–0.03†	[–0.06, 0.00]	
3	–0.03	[–0.06, 0.01]	–0.01	[–0.03, 0.01]	–0.02	[–0.05, 0.01]	
4 or more	–0.00	[–0.05, 0.05]	–0.03	[–0.06, 0.01]	–0.03	[–0.06, 0.01]	
*Child’s gender*							
Male	0.02^*^	[0.01, 0.04]	0.02^**^	[0.01, 0.03]	0.01	[–0.01, 0.03]	
Female	0.00	[0.00, 0.00]	0.00	[0.00, 0.00]	0.00	[0.00, 0.00]	
*N*	3,533.00		3,384.00		3,422.00		

*Note.* ^†^*p* < .1, ^*^p < .05, ^**^p < .01, ^***^p < .001.

## Discussion

### Income and Age

In this nationally representative longitudinal survey of mothers of young children the results show that the most striking differences in exposures to abuse are by age and income, and that, as per Hypothesis (a) there is social gradient in experiences of domestic abuse. Household income was a better dimension for capturing unequal experiences of abuse across all dependent variables explored in the analysis. Compared to mothers in the highest income households, mothers on the lowest income quintile were far more likely to report experiencing any abuse, and they were particularly more likely (fivefold chance) to experience high-intensity abuse, which in our study was defined as having experienced more types of abuse, more often. This pattern also applied when looking at each type of abuse separately. Income was also picking up a gradient effect, with exposure to abuse increasing with decreasing income. These findings resonate with recent data from the Crime Survey for England and Wales which also found abuse prevalence to be the highest among women in the lowest household income bracket and that prevalence of domestic abuse in the last 12 months for women declined as income increased (Office for National Statistics, 2018). Overall, income poverty is consistently reported as a key sociodemographic predictor of domestic abuse (Capaldi et al., 2012; Fahmy et al., 2015; Reis, 2018).

In terms of coefficient sizes, age appeared to be the second most important dimension in predicting unequal exposure to any abuse, to abuse intensity, and to different types of abuse. While there is a gradual increase in odds of abuse exposure with decreasing age, the only statistically significant difference is between the youngest mothers and the oldest mothers. These age related findings are in line with data from recent Crime Survey for England and Wales data (Office for National Statistics, 2018) and with most domestic abuse prevalence literature (see Capaldi et al. 2012, for a review of 228 studies), most of which finds that age is a protective factor in terms of domestic abuse experiences. There could be multiple explanations for this trend. On the one hand younger age at childbirth is correlated with other characteristics which are in turn also correlated with a higher chance of experiencing abuse (such as income poverty). On the other hand, even controlling for these effects, older mothers might have characteristics which deter a potentially abusive partner, such as more social networks, self-confidence, self-belief or assertiveness, although this could be as likely linked to the characteristics of male partners of older mothers. This issue is one which warrants further study.

### Maternal Social Class and Maternal Education

Aside from income, other measures of social stratification were less significant and less straightforward to interpret. Maternal social class showed differences in exposure only when comparing those with semi routine and routine occupations to mothers in managerial and professional occupations, and still the effects size was relatively small. After controlling for confounders, maternal education was perplexing, since it appeared that the odds of exposure to abuse increased with increasing education, thus representing a trend in the opposite direction to that expected given the income and social class findings. Controlling for social class, household income and age, all below degree-level educated mothers were less likely to experience any type of violence than mothers with degrees. The significant interaction effects we found confirm that the combination of being in the poorest household income groups and also having degree or above level education was associated with a much higher predicted prevalence of reported abuse. It is not clear why this might be the case. In line with the relative resource theory of violence, there seems to be some evidence that women with higher educational qualifications than their partners are at a higher risk of abuse (Vyas & Watts, 2008), though in our analysis we see that the effect persists even after controlling for key socioeconomic characteristics of partners. One hypothesis is that, since the model is already controlling for other correlates of maternal education (i.e., age, maternal social class and household income), the residual effect being picked up by the education variable is showcasing a reporting bias effect, that is, that mothers with higher educational qualifications are also more likely to recognize abuse as such, and more likely to report it in the survey. But this is only a hypothesis. In the existing literature, it is unusual to see discussions of several measures of social inequality within a single study, and studies rarely compare the ability of different socioeconomic variables to tease out social inequalities in the experiences of abuse (Capaldi et al., 2012; Jasinski, 2004). Literature reviews looking across studies, seem to concur that unemployment and low income were better and more robust predictors of interpersonal violence, and income was also a better predictor than measures such as education (Capaldi et al., 2012).

The above results stress that what *does* correlate strongly with domestic abuse is poverty, and more specifically income poverty. Being financially dependent and constrained puts women in vulnerable situations and limits their options on a practical level (Capaldi et al., 2012; Reis, 2018). Financial insecurity and dependency make leaving an abusive partner all the more difficult (Fahmy et al., 2015; Postmus et al., 2020).

### Age, Poverty, and Intersectionality

The significant interaction effects found point to the importance of understanding how experiences of domestic abuse, as with other adverse outcomes, are best understood by taking into account how dimensions of disadvantage overlap and interlock with each other. This is the essence of the theory of intersectionality originally introduced by Crenshaw (1989), and which has also been applied to domestic abuse research. Existing studies looking at domestic abuse through an intersectional lens have mostly looked at the interaction of poverty and race, particularly in terms of poor black women’s experiences of domestic abuse in a US context (Bograd, 1999; Conwill, 2010; Nixon & Humphreys, 2010; Sokoloff, 2004; Sokoloff & Dupont, 2005). In the context of our study, the experience of these women is not that they are either poor or young, but that they can often be both poor and young, and the chances of experiencing abuse are therefore compounded, in line with our Hypothesis (c). The highest predicted prevalence of experiencing any domestic abuse was for mothers who were *both* in the youngest *and* the poorest groups in the sample. Among this group it is predicted that 1 in 3 experience some form of abuse. By contrast, 1 in 10 mothers who were neither in the youngest nor poorest categories are predicted to experience some form of abuse.

### Child Sex

Being the mother of a male study child was associated with a small but statistically significant higher chance of experiencing violence than being the mother of a female study child. While there is a vast literature on how children’s sex interacts with children’s outcomes for those who have grown up with domestic abuse, there is little to no evidence on whether male perpetrators are more likely to be abusive towards mothers of male children. In fact, the sex of children of mothers experiencing violence is rarely discussed in research, even in studies where children’s sex is controlled for in regression models, coefficients are occasionally not shown or discussed, possibly due to no significant differences being found (Capaldi et al., 2012; Jasinski, 2004). Some evidence on stepfathers suggests that they are more likely to be abusive to both mothers and their nonbiologically related children due to evolutionary principles related to reproduction and mate guarding (Archer, 2013). However, the increased risk violence towards mothers of male stepchildren compared to female stepchildren may not be adequately explained by such theories.

### Differences Between Types of Domestic Abuse

Overall, the most commonly experienced forms of abuse were coercive control (11%), followed by physical violence (7%) and threats (7%). In line with our Hypothesis (b), differences in prevalence patterns of different types of abuse suggest that experiences of either coercive control, threats or physical abuse, are all stratified by the same variables, in the same “direction,” and with effects of similar magnitude. More coefficients reached statistical significance for coercive control, most likely due to the larger proportion of mothers reporting this form of violence as opposed to for threats or physical abuse (so, a larger subsample). The distinction of abuse types reflects the sets of questions in the survey, where threats were asked about separately, and conceptualized as being different to other questions roughly understood as psychological abuse or coercive control. One could argue that threats too are part of coercive control. GUS questions were originally based on the questionnaire in the Scottish Crime and Justice Surveys, though the latter are about to change significantly to move away from an approach focusing on numbers of incidents of violence to an approach focusing on patterns of behavior of perpetrators and of abusive relationships (M. Scott, personal communication, January 27, 2020).

Generally, studies on large nationally representative surveys looking at different forms of abuse are few and far between, and none have been found to be closely comparable to this study (Alhabib et al., 2010; Capaldi et al., 2012; Jasinski, 2004). The aforementioned review by Capaldi et al (2012) identifies only two out of 228 studies which actually compare different types of abuse by demographic factors, but these are not comparable in terms of their substantive focus (one focuses on female perpetrators in the armed forces, and the other on adolescent dating violence). In a review of global prevalence studies on domestic violence, out of 155 studies included, 88 were population-based studies; and of these 24 asked about physical, sexual, and emotional abuse, and of these, 8 used a random sample greater than 1,000 participants (Alhabib et al., 2010). Looking at more closely comparable data with respect to our study, The Millennium Cohort Study, a very large UK-wide birth cohort study with a similar study design to GUS, asks only one question of mothers on domestic abuse, which focuses exclusively on physical violence, not coercive control (Jofre-Bonet et al., 2016). This data gives an estimated prevalence of domestic abuse of under 4% across three separate waves when this was asked. This disparity in prevalence of abuse when using a narrow versus a broad definition, shows that only collecting information on physical violence vastly underestimates the prevalence of abuse.

### Limitations

There are some study limitations worth summarizing here. As with any questionnaire, or indeed any study exploring a sensitive topic, we do not know the extent to which answers about domestic abuse experiences were accurate, and if the calculations about prevalence are therefore representative of “true” prevalence in the population. It could be that mothers were unable to report accurately (since they were asked to recall over a 6-year period), or intentionally refused to disclose such incidents. We also do not know if there is nonrandom pattern to mothers who intentionally do not disclose abuse happening, that is, if some groups of mothers are more likely to not answer truthfully than others. What we do know is that there is a nonrandom pattern of item nonresponse for the domestic abuse questionnaire module. In analysis not shown, we find that 59 mothers chose not to respond to any domestic abuse questions, either because they forgot or they refused to answer. The item nonresponse rate was 3.5% among the lowest income group, 0.3% among the highest income group, and 6.3% among those with missing income information. This suggests that there is a social gradient to our missing information, and so if anything, this means that both our overall prevalence estimates may be lower than reported here, and the actual gradient of social inequality in abuse experiences may be steeper in the actual population.

Another limitation we wish to discuss concerns our variable on “abuse intensity.” Changes in discourse (Myhill, 2017) and legislation around how abuse is conceptualized have also led to changes in how abuse is measured in leading social surveys, such as the Scottish Crime and Justice Survey. The most recent iteration of the latter survey is being reviewed so that questions on domestic abuse move away from thinking about “incidents” of abuse, towards thinking about abusive patterns of behavior (M. Scott, personal communication, January 27, 2020). To illustrate with a hypothetical example, one could imagine a case where a mother has only been threatened once, but that the severity of this threat within the context of a specific relationship is serious enough for the mother to live with the fear of abuse on a daily basis. Thus, counting incidents runs the risk of not recognizing the nature of abusive relationships and the ongoing impact of these lived experiences of victims (Myhill & Hohl, 2019). We recognize therefore that there is an inherent flaw within our variable of abuse intensity as formulated above. Despite these limitations, we hope that on a collective level (even if not on an individual level), useful insights could be drawn by contrasting the two halves of a spectrum of abuse experiences using simple indicators such as “different types of abuse” and “frequency of abuse.” Ultimately, even if thinking in terms of patterns of abusive behavior (rather than incidents), it would be difficult to define a “pattern” without any discussion of incidents or frequency (Walby & Towers, 2018), though perhaps questionnaires need to be more attuned to the fact that feeling under constant threat of abuse is part of being a victim of abuse, even if this is hard to pin down to specific incidents.

## Conclusion

There are some relevant policy implications which our research brings to light. Firstly, we need to be open to discussions about how we define and measure the different forms that domestic abuse may present in, and to think about the incidence and frequency as being important, but that our primary concern should be about understanding the impact. Next, we have an increasing understanding of how financial insecurity is closely linked with experiences of abuse, so policy makers should take into account how current social and economic policies, and the relationship with income-related and welfare benefits may “trap” women in relationships with abusive partners, and reduce their space for action (Sharp-Jeffs et al., 2018). For example, the current and relatively new universal credit payment system in the UK is meant to help people on low or no incomes manage their living costs. At the time of writing, the system expects claimants who are living together to make one single claim paid out to one claimant on behalf of the couple. As recently noted by a UK parliamentarian (Buchan, 2020), this puts women experiencing domestic abuse at more risk since it potentially makes it easier for a perpetrator to control all couple finances, and in turn makes it harder for women to exit an abusive relationship. Also, the current COVID-19 pandemic is revealing and intensifying existing social inequalities, with particularly serious implications for women experiencing, and at risk of, domestic abuse (Evans et al., 2020). Finally, there is an increasing recognition that for many young people, their first intimate relationships in adolescence are marked by high levels of abuse and violence (Barter et al., 2017). This may account in part for our findings about the greater risk to younger mothers of young children. This reinforces the need to provide robust preventative education to young women and men about respectful relationships (Stanley et al., 2015).
